# Bioactive Components in Whole Grains for the Regulation of Skeletal Muscle Function

**DOI:** 10.3390/foods11182752

**Published:** 2022-09-07

**Authors:** Qing Li, Haihong Yang, Shuimiao Song, Jie Liu, Ziyuan Wang, Jing Wang

**Affiliations:** China-Canada Joint Lab of Food Nutrition and Health, Beijing Technology & Business University, Beijing 100048, China

**Keywords:** whole grains, bioactive components, skeletal muscle function, regulatory mechanism

## Abstract

Skeletal muscle plays a primary role in metabolic health and physical performance. Conversely, skeletal muscle dysfunctions such as muscular dystrophy, atrophy and aging-related sarcopenia could lead to frailty, decreased independence and increased risk of hospitalization. Dietary intervention has become an effective approach to improving muscle health and function. Evidence shows that whole grains possess multiple health benefits compared with refined grains. Importantly, there is growing evidence demonstrating that bioactive substances derived from whole grains such as polyphenols, γ-oryzanol, β-sitosterol, betaine, octacosanol, alkylresorcinols and β-glucan could contribute to enhancing myogenesis, muscle mass and metabolic function. In this review, we discuss the potential role of whole-grain-derived bioactive components in the regulation of muscle function, emphasizing the underlying mechanisms by which these compounds regulate muscle biology. This work will contribute toward increasing awareness of nutraceutical supplementation of whole grain functional ingredients for the prevention and treatment of muscle dysfunctions.

## 1. Introduction

Whole grains refer to the whole, milled into powdered, fragmented or pressed pieces of cereal glume, whose proportion of endosperm, germ and bran is essentially the same as that in intact glume [1]. It has become evident that whole grains and whole-grain-based products possess enormous nutritional and health-promoting properties due to the germ and bran fractions, which contains unique bioactive components (dietary fiber, β-glucan, phenolics, carotenoids and tocotrienol) compared to refined grains [2,3]. Scientific studies showed that the components derived from whole grains exhibit positive modulation on skeletal muscle function. Based on established research, polyphenols extracted from wheat bran, such as alkylresorcinol [4] and ferulic acid [5], could alleviate muscle atrophy caused by obesity. The intake of more dietary fiber is beneficial for promoting muscle mass by improving the composition and metabolic capacity of the gut microbiota [6]. This evidence indicates the potential benefit of bioactive components in whole grains on skeletal muscle function. In addition, the emergence of sports nutrition foods demonstrates the beneficial effect of bioactive components in whole grains. Sports nutrition supplements with bioactive ingredients such as γ-oryzanol and octacosanol have gained increased attention in the functional food market and attracted growing interest especially in the elderly population and athletes [7]. For instance, γ-oryzanol has been commercially approved for enhancing the physical fitness of athletes, used to improve capacity to resist muscle fatigue and enhance muscle strength [8]. Functional drinks and capsules supplemented with octacosanol (0.07 mg/mL and 51 mg/kg, respectively) are shown to exert anti-fatigue, reaction sensitivity improvement and physical recovery effects on athletes [9].

**Figure 1:**
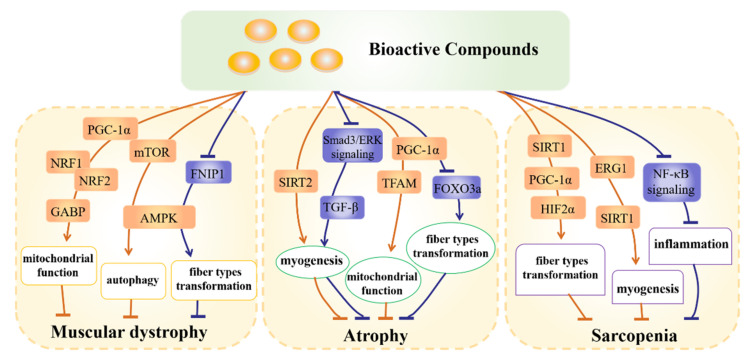
Signaling pathways involved in the regulation of skeletal muscle diseases. → indicates promotion; T indicates inhibition.

Skeletal muscle dysregulation occurs in chronic syndrome caused by obesity and aging, which is characterized by inflammatory reaction, poor mitochondrial function and muscle protein loss [10]. Muscle tissue function depends on the growth and differentiation of myotubes, the transformation of muscle fiber types and mitochondrial function [11,12,13]. The reduction in myotube size will lead to skeletal muscle atrophy, while the improvement in muscle fiber cross-sectional area (CSA) is positively associated with muscle strength and mass [14]. Generally, skeletal muscle is composed of slow-twitch myofibers (types I and IIa) and fast-twitch myofibers (types IIb and IIx). These two muscle types are the basis of muscle plasticity in response to various functional demands [15]. The change in isoforms of myosin heavy chain (MyHC) serves as an important sign for muscle-fiber-type transformation [16]. Exercise and disuse of muscle could stimulate muscle-fiber-type transformation [17]. In addition, mitochondria are involved in the regulation of muscle metabolic function through adaptive changes in mitochondrial quantity, structure and quality [18]. The occurrence of muscle dysfunction is found to be directly related to the impairment of the mitochondrial network [19]. Taken together, these factors mentioned above have been implicated in the pathogenesis of muscle-related diseases, including muscular dystrophy, atrophy and aging-related sarcopenia (Figure 1).

Muscular dystrophy (MD) is characterized by atrophy and weakness of muscle tissue, resulting in degeneration and gradual aggravation of muscles controlling movement. Mitochondrial biogenesis dysfunction and decreased ATP production are important inducements of muscular dystrophy [20]. AMPK has emerged as a key regulator of muscular dystrophy by regulating mitochondrial autophagy and biogenesis-related proteins including PGC-1a, NRF1, NRF-2/GABP and SIRT1 [21]. Multiple regulators can modulate skeletal muscle metabolism through activation of AMPK among which FNIP1 is a key regulatory protein that regulates mitochondrial function, muscle-fiber-type transformation and skeletal muscle health [22]. FNIP1 knockdown could lead to the activation of AMPK and enhancement of mitochondria number, alleviating the symptom of muscular dystrophy [23]. In addition, the decrease in autophagy flux could also cause muscular dystrophy, while AMPK-activated autophagy is reported to be beneficial for improving myofiber structure and function in mice with muscular dystrophy [24]. Moreover, AMPK could also be modulated by mTOR signaling via phosphorylation, which inhibits autophagosome-wrapping-damaged organelles and the process of lysosome-caused degradation [25].

Skeletal muscle atrophy is the consequence of muscle protein degradation exceeding protein synthesis [26]. The destruction of myogenesis and decrease in CSA caused by excessive autophagy are two main reasons for muscle atrophy. SIRT2 is reported to modulate skeletal muscle atrophy by inhibiting excessive autophagy [27] and improve myotube differentiation and hypertrophy [28]. Enhanced mitochondrial function is conducive to improving muscle atrophy [29], while the reduction of mitochondrial biogenesis and oxidative phosphorylation leads to the production of ROS, which causes excessive muscle protein degradation [30]. Mitochondrial transcription factor A (TFAM) binds and coats mtDNA, protecting it from ROS-induced degradation and maintaining mitochondrial function [26]. On the other hand, muscle atrophy is accompanied by slow-to-fast shift in muscle fiber type [31]. The overexpression of FOXO3a could stimulate this process, while PGC-1α could alleviate muscle atrophy through inhibiting the expression of FOXO3a [31]. In addition, TGF-β is mediated by Smad3/ERK-dependent signaling to regulate myotube proliferation and differentiation. It has been reported that TGF-β could inhibit the level of myosin heavy chain (MyHC) and decrease muscle CSA, resulting in skeletal muscle atrophy [32].

Sarcopenia is characterized by a loss of muscle mass and function that reduces mobility. As reported, aging-induced sarcopenia is characterized by the preferential loss of fast-twitch muscle fibers and transformation from type II (fast-twitch muscle fibers) to type I muscle fibers (slow-twitch muscle fibers) [33]. The dysfunction of myotube regeneration and lysosomal degradation are also involved in sarcopenia [34]. It has been proven that activation and overexpression of SIRT1 reduces skeletal muscle aging. SIRT1 directly phosphorylates and enhances the activity of PGC-1α, which targets the hypoxia-inducible factor 2 α (HIF2α) to control muscle fiber transformation [35]. In addition, early growth response 1 (EGR1) has been reported to bind with SIRT1 to improve oxidative-stress-impaired myotube differentiation [36]. Systemic inflammation is one of the main causes of skeletal muscle loss and sarcopenia. For instance, NF-κB signaling is activated in aging people which induces an inflammatory effect and the production of ROS in mitochondria. Moreover, excessive autophagy is aggravated by NF-κB signaling, which causes the degradation of muscle protein [37].

Emerging evidence suggests that nutritional intervention is helpful to improve myotube proliferation, regulate myofiber-type transformation and prevent or delay muscle protein degradation, benefiting muscle mass and metabolic function. In this review, we summarize the bioactive components derived from whole grains (Table 1) and explore the potential role of whole grain bioactive components in improving skeletal muscle function, and further discuss the possible regulatory mechanisms.

## 2. Whole Grain Bioactive Compounds in the Regulation of Skeletal Muscle Function

**Figure 2:**
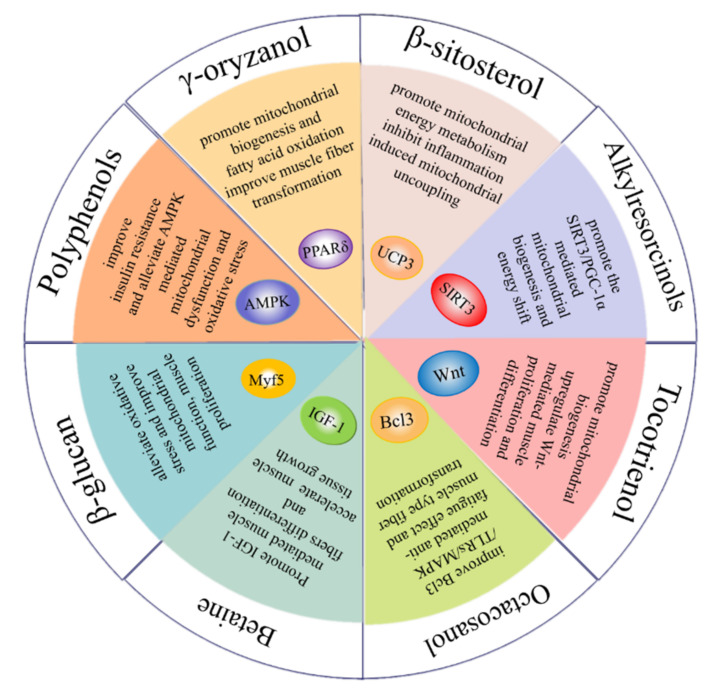
Bioactive components of whole grains for improving skeletal muscle function.

Whole grain consumption has been demonstrated to have an impressive effect on chronic disease prevention, such as type 2 diabetes, cardiovascular disease, cancer and muscle disease associated with obesity and aging [1,38]. Herein, we discuss the evidence which supports the role of whole grain functional ingredients in regulating muscle health through their ability to promote myogenesis, affect muscle-fiber-type conversion, reduce muscle protein loss and enhance muscle metabolic function (Table 2 and Figure 2).

### 2.1. Phenolic Compounds

#### 2.1.1. Phenolic Acids

Ferulic acid is a phenolic acid widespread in whole grains such as oat [39], wheat [40], rice [41], quinoa [42] and corn [43], which is evidenced to possess an impressive effect on modulating muscle function. It has been reported to prevent insulin resistance in skeletal muscle [44]. It can also promote glucose uptake and regulate fatty acid oxidative decomposition in a dose-dependent manner, promoting redox balance in rat muscle [5]. By triggering the PI3K/CpkC pathway, ferulic acid is proved to promote glucose uptake and glycogen synthesis, protecting against insulin resistance in L6 myotubes [45]. After 30 days of dietary ferulic acid supplementation, the number and CSA of muscle fiber are enhanced in a zebrafish model. Ferulic acid also accelerated the muscle tissue growth of zebrafish by upregulating the expression of MyoD and myogenin [46]. In addition, ferulic acid is proven to modulate PGC-1α-activated mitogenesis and energy metabolism via upregulating the expression of NRF1, TFAM and TFB2M [47]. In C2C12 cells, ferulic acid exerts an anti-fatigue function via increasing succinate dehydrogenase (SDH) activity and decreasing lactate dehydrogenase (LDH) activity. Moreover, ferulic acid could stimulate the transformation from fast-twitch muscle fibers to slow-twitch muscle fibers via the SIRT1/AMPK signaling pathway [48]. This effect is further verified in a weaned piglets model, demonstrating that SIRT1/AMPK/PGC-1α signaling was involved in ferulic-acid-mediated muscle transformation and anti-fatigue function [49]. This evidence demonstrates the positive effect of ferulic acid on muscle metabolism and function.

*p*-coumaric acid is widely distributed in whole grains including rice [50], quinoa [42], barley [51] and wheat [40]. It has been found that *p*-coumaric acids possess the ability to regulate myogenic differentiation and lipid metabolism of skeletal muscle. For instance, *p*-coumaric acid could increase the expression of myogenic regulatory factors myogenin and MyoD via activating AMPK signaling, improving myogenic differentiation of C2C12 cells [52]. In the L6 cell model, *p*-coumaric acid could increase fatty acid β-oxidation through stimulating ACC phosphorylation and the expression of PPARγ, preventing skeletal muscle against oxidative stress and inflammation caused by fatty acid accumulation [53].

#### 2.1.2. Resveratrol

Resveratrol is a natural source of antioxidant and mitochondrial nutrients which can be found in whole grains, such as buckwheat [54]. Resveratrol shows a positive effect on mitochondrial-dysfunction-induced muscle atrophy. For instance, in a dexamethasone-induced mouse model, resveratrol alleviates dexamethasone-induced mitochondrial dysfunction and muscle atrophy by blocking AMPK/FOXO3 signaling [55]. In high-fat-diet-induced aged mice, muscle loss and myofiber size decrease are reversed by resveratrol supplementation. Resveratrol could improve the mitochondrial dysfunction and oxidative-stress-induced muscle atrophy via activation of the PKA/LKB1/AMPK pathway [56]. Moreover, resveratrol has been reported to enhance muscle proliferation and differentiation in obese mouse muscle. The level of muscle regeneration proteins including MyoG, Myf5 and Pax7 is upregulated by resveratrol administration [57]. In response to skeletal-muscle glucose metabolism disorders caused by obesity, resveratrol can reduce insulin resistance by increasing muscle glycogen synthesis and reducing ROS levels in high-fat-diet-fed mice [58]. In the C2C12 model, resveratrol promotes AKT activation and glucose absorption, as well as decreased glutathione (GSH) level. These results suggest that resveratrol could alleviate muscle insulin resistance by modulating redox levels [59]. Resveratrol is evidenced to alleviate TNF-α-induced muscle hypertrophy and muscle atrophy in C2C12 cells via inhibiting the atrophy-related ubiquitin ligase through upregulating AKT/mTOR/FOXO1 signaling [60]. Furthermore, resveratrol displays impressive activity in promoting the transformation of muscle fiber types. The expression of myosin heavy chain (MyHC) 1, MyHC2a and MyHC2x in mouse muscle is increased with resveratrol supplementation, indicating the transformation from fast- to slow-twitch muscle fibers. This conversion is achieved by activating the AdiopR1-AMPK-PGC-1α signaling pathway [61]. In C2C12 myotubes, resveratrol supplementation increased the activities of lactate dehydrogenase (LDH) and malate dehydrogenase (MDH) while it reversed the elevated level of miR-22-3p, suggesting its anti-fatigue effect via stimulating the transformation of muscle fiber from fast-twitch to slow-twitch type [62]. In addition, resveratrol exerts an anti-fatigue effect in contusion-induced-injury mice, shown as the increased activity of lactate dehydrogenase (LDH) and creatine kinase (CK), displaying an important role in muscle metabolic regulation [63].

#### 2.1.3. Flavonoids

As a special subclass of flavonoids, quercetin is a natural bioactive compound abundantly present in common cereal grains, especially in buckwheat [64], followed by rice [50], quinoa [42], corn [43] and oat [39]. Quercetin has displayed notable antioxidative and anti-inflammatory effects and shows potential benefits for muscle function [65]. By increasing the expression of adiponectin, quercetin stimulates muscle-fiber-type transformation from fast-twitch to slow-twitch myofibers [66]. It has been also demonstrated that quercetin increases slow-twitch myofibers by regulating AMPK/SIRT1 signaling [67]. Heme oxygenase-1 (HO-1) serves as an essential factor to decrease the inflammatory response and oxidative stress. Mediated by an HO-1/NRF2-dependent mechanism, quercetin is found to alleviate obesity-induced muscle atrophy [68]. In relation to the mitochondrial network, quercetin has been shown to significantly improve mitogenesis in denervated mice, through which muscle atrophy is improved [69]. Moreover, it is shown that quercetin ameliorates TNF-α-induced skeletal muscle insulin resistance in C2C12 cells [70]. The anti-fatigue function of quercetin might be related to its ability to scavenge free radicals [71]. Overloaded exercise causes excessive ROS generation, which further leads to mitochondrial dysfunction and muscle protein loss [72]. In a BALB/c mouse model, quercetin displayed obvious fatigue resistance activity, shown as enhanced muscle glycogen content, elevated mitochondrial fatty acid β-oxidation and decreased oxidative stress [73]. These findings demonstrate the impressive effect of quercetin on improving muscle mitochondrial function and alleviating the inflammatory reaction in the muscle.

Oligomeric procyanidins (OPCs) are mainly found in dark-colored grains, such as black rice and red rice [74]. As reported, OPCs could effectively inhibit glucose metabolism disorder in muscle tissue caused by diabetes. OPCs are shown to improve lipid metabolism by inhibiting mTOR signaling and enhancing glucose homeostasis and insulin sensitivity in the skeletal muscle of diabetic mice [75]. In obese mice, OPC supplementation increases glycogen synthesis and glucose uptake, exerting an antidiabetic effect in an insulin-independent manner [76]. Moreover, oral OPC administration alleviates acute hyperglycemia and improves insulin sensitivity via promoting AMPK-signaling-mediated GLUT4 translocation in ICR mice [77].

Cyanidin-3-glucoside (Cy3G) widely exists in pigmented whole grains, such as black rice [50], purple corn [43], oat, wheat and rye [78]. In vivo and in vitro studies have shown that Cy3G possesses anti-obesity and antidiabetic effects [79,80]. It has been demonstrated that as effective radical scavengers, Cy3G exerts an anti-diabetes effect via reducing oxidative stress in human skeletal muscle cells [81]. In an ICR mouse model, oral supplementation with Cy3G shows improved exercise endurance, performed as weight-loaded swimming time. Moreover, the increased level of lactic acid accumulated after excessive exercise is reversed by Cy3G via activating PGC-1α signaling, indicating that Cy3G exerts its anti-fatigue effect through modulating the PGC-1α-mediated lactic acid metabolism [82]. Consistent with the in vivo data, Cy3G could also upregulate PGC-1α expression in C2C12 myotubes, improving muscle metabolic function [82].

Catechins are flavanols widely found in whole grains such as wheat [83], barley and buckwheat [84]. The isomers of catechins including catechin, epicatechin (EC) and epigallocatechin gallate (EGCG) are proved to increase muscle mass and muscle strength, enhancing the exercise endurance of rats [85]. Catechins could upregulate the expression of Myf5 in muscle stem cells via activating Akt phosphorylation and thus improve muscle regeneration [86]. In the C2C12 cell model, catechin supplementation was found to promote myotube differentiation, shown as an increased level of MyoD, MyoG and MyHC, suggesting its improvement effect on skeletal muscle regeneration and repair [87]. Moreover, catechins have shown impressive modulation of the promotion of mitochondrial function in skeletal muscle. In an LCR (low running capacity) rat model, EC treatment improved mitochondrial biogenesis and respiratory capacity via activating p38 MAPK signaling in the skeletal muscle, enhancing muscle resistance to fatigue [88]. Using a diabetic rat model, EGCG supplementation could alleviate diabetes-induced mitochondrial dysfunction in skeletal muscle, characterized by increased expression of PGC-1α, MFN2 and COXⅠ. The improvement effect of catechins is possibly achieved by promoting mitochondrial autophagy via activating the ROS-ERK/JNK-p53 pathway [89]. Moreover, EGCG could target Wnt signaling and increase the level of downstream MyoD, promoting muscle regeneration [90]. In a rat model of sarcopenia, EGCG administration could increase skeletal muscle mass which is presumably attributed to the downregulated muscle protein degradation mediated by the ubiquitin–proteasome system (UPS) and the upregulated muscle protein synthesis mediated by IGF-1 [91]. Moreover, EGCG inhibits the expression of oxidatively modified protein induced by electrical stimulation in rat skeletal muscle, preventing the muscle from oxidative damage [92]. These data suggest that catechin could facilitate skeletal muscle function through promoting myotube differentiation, enhancing mitochondrial function and defending oxidative stress.

Rutin is a flavonol glycoside and is widely distributed in whole grains such as buckwheat [84], wheat [93] and quinoa [94]. The antioxidant and anti-inflammatory properties of rutin are demonstrated from both in vivo and in vitro studies [95]. The decreased mitochondrial number and mitochondrial biogenesis in skeletal muscle are associated with obesity [96]. In an obese rat model, rutin supplementation significantly enhances mitochondrial DNA (mtDNA) content and mitochondrial biogenesis in skeletal muscle by activating AMPK signaling. The expression levels of PGC-1α, NRF1, TFAM and SIRT1 are also increased, indicating that rutin could restore obesity-induced skeletal muscle mitochondrial dysfunction [97]. Using a forced swimming mouse model, rutin administration was proved to upregulate PGC-1α-mediated mitochondrial biogenesis and decrease the level of lactic acid in skeletal muscle, improving the fatigue-resistance capacity of mouse muscle [98]. In addition, rutin was found to inhibit oxidative stress and inflammation-induced muscle injury. Specifically, rutin intervention improves the inflammatory state by inhibiting NF-κB signaling, manifested in the decreased expression of IL-6 and iNOS, preventing muscle injury of C2C12 cells [99].

### 2.2. Carotenoids

Carotenoids are lipid-soluble phytochemicals and the inactive precursor of vitamin A [100], which are abundantly distributed in corn, wheat and barley [101]. As the main component of carotenoids, lutein is reported to improve muscle function via alleviating oxidative stress [102]. In a rat model of ischemia-reperfusion (IR) muscle injury, lutein supplementation decreases the production of ROS and the expression of COX-2 by inhibiting NF-κB signaling, protecting against skeletal muscle IR injury [103]. These observations imply the muscle improvement benefit of lutein presumably via its antioxidant and anti-inflammatory effect.

β-carotenes are the most ubiquitous and stable natural pigments in nature [104]. β-carotene supplementation could enhance muscle mass by promoting IGF-1-mediated muscle protein synthesis and reducing ubiquitin–protease-mediated muscle protein degradation in an atrophy mouse model [105]. Atrogin-1 and MuRF1 are identified as muscle-specific ubiquitin ligases that are upregulated in skeletal muscle under atrophy-inducing conditions [106]. β-carotene treatment is reported to increase muscle mass and decrease the level of Atrogin-1 and MuRF1 in denervated mice, exhibiting a promotion effect on oxidative-stress-induced muscle atrophy [107].

### 2.3. Tocotrienol

Tocotrienol, the unsaturated form of vitamin E, mainly exists in buckwheat [108], rice, rye and oat [39]. Tocotrienol receives attention due to its unique biological properties such as antioxidant and anti-inflammatory activity [109]. Increasing evidence has shown that tocopherol exhibits a positive effect on maintaining muscle function. It has been proven that dietary supplementation of tocotrienol improves muscle atrophy and insulin resistance in type 2 diabetic mice. This effect is achieved by activating AMPK/SIRT1/PGC-1α signaling and increasing mitochondrial biogenesis in the skeletal muscle, indicating that tocotrienol improves muscle disorder via upregulating mitochondrial function [110]. In addition, muscle differentiation and regeneration are essential for protecting against aging-related loss of muscle mass and strength. Tocotrienol treatment is found to increase the expression of myoblast-differentiation-related proteins such as MyoD, MyoG and MRF4. Moreover, tocotrienol increased the miR-206 level in human skeletal muscle myoblasts (HSMMs), which resulted in an increased expression of IGF1R and decreased expression of Pax7, promoting muscle cell differentiation [111]. Through transcriptomic analysis, tocotrienol was found to promote muscle cell regeneration via Wnt signaling and alleviate aging-related muscle loss through the FOXO pathway [112]. Additionally, Chung et al. reported that tocotrienol administration improved insulin intolerance and increased soleus muscle weight of obese mice, indicating its potential to ameliorate muscle dysfunction [113].

### 2.4. β-Glucan

β-glucan is a soluble dietary fiber which exists abundantly in oat [39], rice [114], wheat, barley and rye [108]. β-glucan has been evidenced to have a positive effect on muscle health [115]. For example, dietary supplementation with oat β-glucan is found to decrease the activity of lactate dehydrogenase and creatine kinase in serum, while it increases the glycogen content in the gastrocnemius muscle of rats. These results indicate that oat β-glucan facilitates the recovery of trained rats from fatigue [116]. In addition, β-glucan is found to promote the transformation of fast-twitch muscle fibers to slow-twitch type in the C2C12 model. Moreover, the levels of myoblast proliferation markers such as Myf5 and Mox2 are increased by β-glucan treatment, suggesting the anti-fatigue effect via improving muscle fiber proliferation and transformation [117]. Mitochondria are responsible for providing energy during immune action and the enhancement of mitochondrial function is conducive to preventing oxidative stress. In the Duchenne muscular dystrophy (DMD) zebrafish model, β-glucan intervention improves mitochondrial respiration and zebrafish exercise capacity, suggesting that β-glucan could alleviate the symptoms of DMD through regulating mitochondria function [118].

### 2.5. γ-Oryzanol

γ-oryzanol is a mixture of feruloyl esters with triterpenol as the main component and is mainly found in rice, corn and wheat [119,120]. γ-oryzanol supplementation has been evidenced to improve exercise performance such as grip strength and running time in aged mice. Additionally, γ-oryzanol has been found to promote the formation of slow-twitch muscle fiber, which is a fatigue-resistant myofiber type and conductive to the prevention of muscle aging. By binding to PPARδ and ERRγ directly, γ-oryzanol could upregulate mitochondrial biogenesis and alleviate inflammation, reducing the occurrence of muscle weakness of the aged mice [121]. Moreover, γ-oryzanol supplementation is demonstrated to promote GLUT4 translocation, alleviating insulin resistance in obese mice [122]. In addition, the antioxidant function of γ-oryzanol in muscle could be enhanced under an exercise condition [123], suggesting the physical exercise combined with γ-oryzanol supplementation might be a beneficial strategy to prevent muscle dysregulation.

### 2.6. β-Sitosterol

As the most abundant phytosterol, β-sitosterol exists widely in plant products such as barley [51], wheat, oat and quinoa [39,124]. It is considered to be a mild free-radical scavenger and has positive effects on maintaining cell membrane stability, as well as inhibiting inflammation and Alzheimer’s disease [125]. In ICR mice, β-sitosterol promotes mitochondrial electron transport and mitochondrial oxidative phosphorylation, enhancing muscle strength. In addition, β-sitosterol promotes the energy metabolism of C2C12 cells by activating UCP3 and inducing mitochondrial uncoupling [126]. Meanwhile, β-sitosterol dramatically enhanced mitochondrial biogenesis in chicken skeletal muscle via activating the PGC-1α/TFAM pathway [127]. In an L6 cell model, β-sitosterol could promote glucose uptake and lipid metabolism by activating ACC phosphorylation. A decreased level of triglycerides and cholesterol is achieved with LKB1-mediated AMPK activation [128]. Likewise, β-sitosterol supplementation is proved to facilitate GLUT4 translocation and upregulate insulin sensitivity in HFD-induced diabetic rats, manifested in the increased activity of glycolytic and gluconeogenesis enzymes [129]. Taken together, these data suggest the promoting effect of β-sitosterol on mitochondrial function and glucose metabolism in the skeletal muscle.

### 2.7. Alkylresorcinols

Alkylresorcinols (ARs) are phenolic lipids and exist abundantly in the bran part of cereal grains such as wheat, barley and rye [108]. They consists of various homologues distinguished by the length of the alkyl chain (C17:0−C25:0) attached to the phenyl ring [3]. ARs derived from wheat bran extracts have been indicated to prevent muscle atrophy in denervated mice via regulating the disruption of fatty acid metabolism induced by lipid autophagy [4]. In addition, dietary supplementation containing ARs was found to protect against isoproterenol-induced myofibrillar degeneration in rats via inhibiting oxidative damage caused by lipid accumulation [130]. 5-heptadecylresorcinol (AR-C17) as the main homologue of ARs has been proven to have the best anti-inflammatory effect among the identified ARs homologues in our previous study [3]. We found that AR-C17 alleviated muscle dysfunction via upregulating SIRT3/PGC-1α-mediated mitochondrial function. The mitochondria content and mitochondrial-biogenesis-related proteins such TFAM and NRF1 were significantly increased, contributing to enhanced exercise performance [131].

### 2.8. Betaine

Betaine is a natural antioxidant widely present in common cereals including whole grain wheat, barley, rye and oat [132]. Betaine supplementation is reported to improve muscle strength and power [133]. IGF-1 stimulates myotube growth and differentiation by activating the expression of downstream MyoD and myogenin. Betaine is reported to increase the expression of IGF-1 in murine myoblasts [134]. AKT is considered as a key regulator of muscle signaling and protein synthesis. Additionally, betaine supplementation could increase anabolism and weaken catabolism in muscle tissue by stimulating IGF-1/AKT signaling [135]. In an in vitro C2C12 model, betaine supplementation promoted myotube differentiation by upregulating the secretion of miR-29b-3p. In addition, through the activation of NFATc1/MyoD signaling, betaine promotes the transformation from fast-twitch to slow-twitch myofibers [136]. Moreover, betaine could promote muscle energy metabolism by upregulating mitochondrial biogenesis, accompanied by increased glucose consumption and ATP production in C2C12 cells [137].

### 2.9. Octacosanol

Octacosanol is a primary aliphatic alcohol that is abundant in rice [138] and wheat [139]. According to literature data, octacosanol has been identified as an anti-fatigue agent which could be stored in skeletal muscle after serial doses administration [140]. In a trained rats model, dietary supplementation with octacosanol also showed a better physical recovery after exhaustion and there was a shift from fast-twitch to slow-twitch myofibers in skeletal muscle [141]. In addition, orally administrated octacosanol might be converted into fatty acids and related to energy utilization, indicating its potential to improve exercise capacity through upregulating the energy supply [142]. The underlying mechanism of the anti-fatigue effect by octacosanol might be through increasing the expression of Prx and decreasing the expression of Trim63 in trained mice. Based on the gene ontology analysis, this improvement might be mediated by the Bcl3/TLRs/MAPK signaling pathway [143]. Overall, octacosanol resists fatigue possibly via modulating muscle-fiber-type transition and muscular energy metabolism.

**Table 1:** The structure and content of bioactive components in whole grains.

Components	Structure	Derived Grains	Content (mg/kg)	References
γ-oryzanol	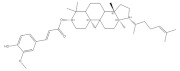	Rice	1550.0–8400.0	[119]
		Wheat	297.0–584.0	[120]
		Corn	200.0–250.0	[120]
β-sitosterol	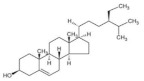	Wheat	365.6–673.1	[124]
		Barley	510.0–676.0	[51]
		Quinoa	76.0–556.0	[39]
		Oat	210.0–409.2	[39]
Alkylresorcinols	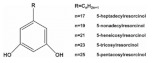			
		Rye	360.0–3200.0	[108]
		Wheat	317.0–1732.0	[108]
Ferulic acid	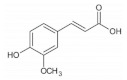	Barley	41.0–210.0	[108]
		Corn	55.2	[43]
		Wheat	472.0–813.8	[40]
		Barley	41.0–210.0	[108]
		Quinoa	126.8–281.7	[42]
		Brown rice	7.1–52.7	[50]
		Rice	155.6–271.1	[41]
		Oat	1493.6	[39]
*p*-coumaric acid	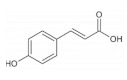	Black rice	84.8 ± 1.4	[50]
		Red rice	176.9 ± 2.5	[50]
		Brown rice	76.1–152.0	[50]
		Wheat	23.8–35.6	[40]
		Barley	0.8–58.4	[51]
		Quinoa	31.3–42.0	[42]
Resveratrol	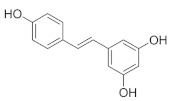	Buckwheat	5.7–7.9	[54]
Quercetin	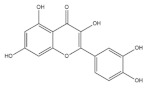	Buckwheat	3700.0	[64]
		Rice	22.0–28.0	[50]
		Barley	1.4–8.7	[51]
		Quinoa	11.3–42.8	[42]
		Corn	15.8	[43]
		Oat	89.0	[39]
Oligomeric procyanidins	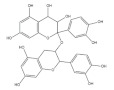	Black rice	3500.0	[74]
		Red rice	200.0	[74]
Cyanidin-3-glucoside	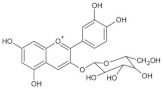	Wheat	1590.0	[78]
		Rice	2682.0–4700.0	[50]
		Purple corn	1430.0	[43]
		Rye	2270.0	[78]
		Barley	1020.0	[78]
		Oat	430.0	[78]
Catechins	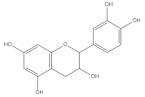	Barley	1312.47 ± 7.11	[84]
		Wheat	355.87	[83]
		Buckwheat	46.47 ± 0.17	[84]
Rutin	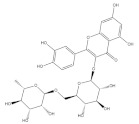	Wheat	236.2	[93]
		Quinoa	609.1	[94]
		Buckwheat	69.95 ± 2.25	[84]
Carotenoids	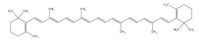	Corn	9.69–13.0	[101]
		Wheat	32.1–39.7	[101]
		Barley	0.15–10.5	[101]
Betaine	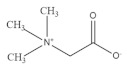	Oat	200.0–1000.0	[132]
		Wheat	270.0–1110.0	[132]
		Rye	444.0–2213.0	[132]
		Barley	460.0–980.0	[132]
Octacosanol	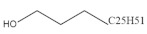	Rice	95.7	[138]
		Wheat	0.4–8.9	[139]
β-glucan	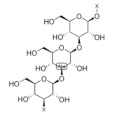	Rice	140.0–570.0	[114]
		Barley	4000.0–7000.0	[108]
		Oat	51,800.0–282,000.0	[39]
		Rye	1200.0–2900.0	[108]
		Wheat	400.0–1400.0	[108]
Tocotrienol	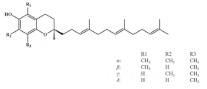	Black rice	31.9 ± 0.4	[50]
		Red rice	36.9 ± 1.6	[50]
		Brown rice	19.6 ± 0.4	[50]
		Wheat	27.81	[108]
		Rye	27.78	[108]
		Barley	18.73	[108]
		Oat	11.59	[108]
		Buckwheat	54.6–552.2	[39]

## 3. Clinical Trials

Whole grains have been shown to possess a range of human health benefits due to their unique health-promoting nutrients in the bran, endosperm and germ tissues [38]. For instance, dietary fiber which is better preserved in whole grains has been reported to prevent age-associated declines in skeletal muscle mass. According to the analysis aimed at adults aged 40 and older in America during 2011–2018, higher dietary fiber intakes are associated with increased muscle strength, as well as improvements in muscle glucose homeostasis [144]. After supplementation with whole grain oats rich in dietary fiber, female individuals are found to have improved exercise capacity, along with increased utilization rate of glycogen and respiratory exchange ratio while decreased free fatty acid content in muscle tissue [145]. In a random trial, dietary intervention with high cereal fiber (whole grain wheat/rye: 30–50 g/day) effectively decreased muscle fat accumulation and improved the situation of insulin resistance and inflammatory reaction in the muscle of T2D patients [146]. Consistently, adopting a healthy diet pattern rich in whole grains is conducive to the improvement in muscle function in adults aged 60 or older, characterized by a higher muscle mass and lower rate of sarcopenia [147]. Interestingly, people with a diet rich in catechins are found to have improved skeletal muscle strength. For instance, 62 males (aged 70) who received an 8-week catechin supplementation (1 mg/kg/d) showed improved muscle strength and decreased expression of MSTN, suggesting the beneficial effect of catechin on aging-related sarcopenia [148]. Moreover, patients with type 2 diabetes who received catechin supplementation (100 mg/d) for 3 months were found to have enhanced mitochondrial biogenesis and reduced mitochondrial damage in the skeletal muscle, alleviating diabetes-induced muscle dysfunction [149]. As a whole grain bioactive component, carotenoids exhibit notable antioxidant and senescence retarding effects and are inversely associated with sarcopenic symptomology [150]. For overweight and elderly people, a diet rich in carotene is conducive to maintaining skeletal muscle function and reducing the development of sarcopenia [151]. In addition, carotene supplementation could decrease the inflammation level and oxidative damage in the muscle of adults aged 65 [152]. Moreover, according to the survey that investigated the effect of carotene on muscle health of 80 adults aged 50 years old, carotene intervention significantly increased muscle insulin sensitivity, suggesting its positive effect on muscle glucose metabolism [153]. In a recent survey, betaine administration also showed an impressive effect on the regulation of skeletal muscle mass. After receiving betaine supplement for 3 years, a significant reduction in muscle loss was observed in the 1242 male participants aged 41–60 [154]. Additionally, adolescent handball players were found with higher exercise endurance and stronger anti-fatigue performance after two weeks of betaine supplementation (2.5 g/day) [155]. Among 23 male subjects, 11 of whom accepted betaine supplementation (2.5 g/day) along with 6-week training showed improved muscle mass and decreased muscle fat accumulation [156]. In summary, these clinical data demonstrate the physiological activity of whole grain components and their regulation effect on skeletal muscle dysfunction.

**Table 2:** Bioactive compounds derived from whole grains for the regulation of muscle function.

Compounds	Experimental Model	Effective Dose	Targets	Target Process	Effect	References
γ-oryzanol	Male C57BL/6 mice	14.78 mg/kg/d	PPARδ ERRγ	Upregulate mitochondrial biogenesis and promote fatty acid β-oxidation	Reduce muscle weakness, alleviate inflammation and enhance muscle strength	[121]
	Male Wistar rats	0.5% (*w*/*w*)	GLUT4	Promote antioxidant and anti-inflammatory effects	Alleviate insulin resistance in muscle	[122]
β-sitosterol	ICR mice C2C12 cells	35 or 350 μg/kg/d 15 μM	UCP3	Upregulate the activity of complex proteins in the mitochondrial electron transport chain and induce mitochondrial uncoupling	Meet energy demand and promote muscle strength	[126]
	Broilers	100 mg/kg	PGC-1α/TFAM signaling	Upregulate oxidative status and mitochondrial biogenesis	Promote muscle performance	[127]
	L6 cells	20 μM	LKB1/AMPK signaling	Increase ACC phosphorylation and triglyceride metabolism	Promote glucose homeostasis and lipid metabolism	[128]
	Male diabetic rats	20 mg/kg/d	Rab/IRAP/Munc 18 pathway	Upregulate GLUT4 translocation and glycolytic and gluconeogenesis enzymes	Alleviate insulin resistance in muscle	[129]
	Male C57BL/6 mice	200 mg/kg/d	FoxO1 signaling	Alleviate muscle protein loss and inhibit protein degradation	Alleviate muscle atrophy	[157]
Alkylresorcinols	Male C57BL/6 mice	0.4% (*w*/*w*)	Pyruvate dehydrogenase kinase 4 (PDK4)	Promote lipid-autophagy-induced lipid metabolism disorder	Inhibit the reduction in muscle size and prevent muscle atrophy	[4]
	Male Wistar rats	400 mg/kg/d	Glutathione peroxidase (GPx)	Inhibit oxidative damage caused by lipid accumulation and increase the expression of lactate dehydrogenase (LDH), alanine transaminase (ALT)	Alleviate myocardial infarction and exert an anti-fatigue effect	[130]
	Male C57BL/6J mice	30 or 150 mg/kg/d	SIRT3/PGC-1α signaling	Increase mitochondrial content and mitochondrial biogenesis	Improve exercise capacity	[131]
Ferulic acid	Male SD rats	0.6 mg/kg/d	PKCε	Inhibit lipid-accumulation-induced inflammation	Alleviate insulin resistance	[44]
	Isolated rat psoas muscle cells from Sprague Dawley male albino rats	15–240 µg/mL	α-glucosidase and α-amylase	Promote fatty acid oxidative decomposition and inhibit carbohydrate and lipid hydrolyzing enzymes	Alleviate oxidative stress and mitigate redox imbalance	[5]
	C2C12 cells	25 μM	PI3K/Cpkc signaling	Promote glucose uptake and glycogen synthesis	Improve insulin resistance	[45]
	Zebrafish	0.06 mg/d	MyoD myogenin	Promote muscle growth	Increase the CSA of muscle fiber and muscle mass	[46]
	Duroc × Landrace × Yorkshire (DLY) weaned piglets	0.05% or 0.45% (*w*/*w*)	SIRT1/AMPK/PGC-1α signaling	Improve the activity of SDH and MDH, increase the expression of slow MyHC protein	Increase the proportion of slow-twitch fiber and promote mitochondria function	[47]
	C2C12 cells	0.5 or 1 μM	SIRT1/AMPK signaling	Increase the protein level of slow MyHC and decrease the protein level of fast MyHC	Promote slow oxidative muscle fiber formation and inhibit fast muscle fiber formation and exert an anti-fatigue function	[48]
*p*-coumaric acid	L6 cells	100 μM	AMPK signaling	Promote the fatty acid β-oxidation	Inhibit lipid-accumulation-induced inflammation in muscle	[53]
	C2C12 cells	0.1 mM	AMPK signaling	Increase expression of myogenin and myoD	Improve myogenic differentiation	[52]
Resveratrol	Male C57 BL/6J mice	50 mg/kg/d	AMPK/ FOXO3 signaling	Improve mitochondrial function	Improve muscle atrophy	[55]
	Male C57BL/6J mice	0.4% (*w*/*w*)	AMPK/PGC-1α signaling	Increase the level of muscle regeneration proteins including MyoG, Myf5 and Pax7 and mitochondrial biogenesis	Enhance muscle proliferation, differentiation and regeneration of impaired muscle	[57]
	L6 cells	25 μM	PKA/LKB1/ AMPK pathway	Improve mitochondrial dysfunction and oxidative stress	Increase muscle mass and myofiber size and improve induced muscle atrophy	[56]
	Male C57BL/6J mice	15 mg/kg/d		Increase muscle glycogen synthesis and reduce ROS levels	Reduce insulin resistance and promote lipid metabolism	[58]
	C2C12 cells	50 μM	AKT signaling	Modulate redox levels and glucose absorption	Reduce insulin resistance	[59]
	C2C12 cells	100 μM	AKT/mTOR/FOXO1 signaling	Inhibit the atrophy-related ubiquitin ligase	Improve muscle hypertrophy and muscle atrophy	[60]
	Male Kunming mice	400 mg/kg/d	AdiopR1–AMPK–PGC-1α signaling	Increase the expression of myosin heavy chain (MyHC) 1, MyHC2a and MyHC2x	Improve the transformation from fast- to slow-twitch muscle fibers and exercise performance	[61]
	C2C12 cells	50 μM	miR-22-3p	Increase the activities of lactate dehydrogenase (SDH) and malate dehydrogenase (MDH)	Promote muscle-fiber-type conversion from fast-twitch to slow-twitch muscle fibers and exert an anti-fatigue effect	[62]
	Male ICR mice	25 mg/kg/d		Increase the activities of LDH (lactic dehydrogenase) and creatine kinase (CK)	Improve muscle recovery and inflammation	[63]
Quercetin	Male Balb/c mice	0.5% (*w*/*w*)	Adiponectin signaling	Decrease the fast MyHC and MyHC IIb protein expression	Promote muscle-fiber-type transformation from fast-twitch to slow-twitch muscle fibers	[66]
	Male C57BL/6 mice	0.05% (*w*/*w*)	HO-1/NRF2 signaling	Decrease inflammatory response and oxidative stress	Reduce obesity-induced muscle atrophy	[68]
	Male C57BL/6 mice	0.2% (*w*/*w*)	PGC-1α signaling	Improve mitochondrial biogenesis and oxidative phosphorylation	Alleviate disuse-induced muscle atrophy	[69]
	C2C12 cells	20 µM	AMPK signaling	Enhance insulin-stimulated glucose uptake and decrease inflammatory response	Ameliorate inflammation-induced insulin resistance	[70]
Oligomeric procyanidins (OPCs)	Male ICR mice	15 mg/kg/d	mTOR signaling	Increase glucose uptake and glycolysis, improve heat generation and inhibit gluconeogenesis and lipogenesis	Improve glucose homeostasis, lipid metabolism and insulin sensitivity	[75]
	Human primary skeletal muscle cells	10 or 25 μM	AKT signaling	Increase glycogen synthesis and glucose uptake	Improve glucose utilization and alleviate insulin resistance	[76]
	Male ICR mice	10 μg/kg/d	AMPK signaling	Promote GLUT4 translocation	Increase insulin sensitivity	[77]
Cyanidin-3-glucoside (Cy3G)	Male ICR mice	1 mg/kg/d	PGC-1α signaling	Improve mitochondrial content and mitochondrial biogenesis	Improve exercise capacity	[82]
	Human skeletal muscle cells	100 µM		Inhibit the activity of α-amylase and α-glucosidase	Alleviate diabetes	[81]
Catechin	C2C12 cells	20 µM	MyoD, MyoG, and MyHC	Promote myotube differentiation	Improve skeletal muscle regeneration and repair	[87]
	Male C57BL/6 mice	25 mg/kg/d	Wnt signaling	Promote myotube differentiation	Promote muscle regeneration	[90]
	C2C12 cells	10 μM	Akt	Promote myotube differentiation	Improve muscle regeneration	[86]
	Male rats	1.0 mg/kg/d	p38 MAPK signaling	Promote mitochondrial respiratory capacity and mitochondrial biogenesis	Enhance the ability of resisting fatigue	[88]
	Male Goto–Kakizaki (GK) rats	100 mg/kg/d	ROS-ERK/JNK-p53 pathway	Promote mitochondrial autophagy	Alleviate diabetic-induced sarcopenia	[89]
	Male Sprague Dawley rats	200 mg/kg/d	IGF-1	Downregulate UPS-mediated muscle protein degradation and upregulate IGF-1-mediated muscle protein synthesis	Increase muscle mass	[91]
	Male SD rats	0.1% (*w*/*w*)		Inhibit the expression of oxidative-modified proteins	Prevent muscle from oxidative stress induced by free radicals	[92]
Rutin	Male Sprague Dawley rats	0.1% (*w*/*w*)	AMPK signaling	Enhance mitochondrial DNA (mtDNA) content and mitochondrial biogenesis	Improve obesity-induced muscle mitochondrial dysfunction	[97]
	Male C57BL/6 mice	60 mg/kg/d	PGC-1α signaling	Upregulate mediated mitochondrial biogenesis and decrease the level of lactic acid	Improve fatigue-resistance capacity	[98]
	C2C12 cells	100 μM	NF-κB signaling	Decrease the expression of IL-6 and iNOS and the production of ROS	Inhibit oxidative-stress-induced skeletal muscle injury	[99]
Lutein	Male Wistar rats	0.5 mg/kg/d	NF-κB signaling	Reduce oxidative stress and inflammation and decrease the production of ROS	Improve skeletal muscle IR injury	[103]
β-carotene	Male Kwl: ddY mice	0.5 mg/kg/d	IGF-1	Promote protein synthesis and reduce ubiquitin-mediated muscle protein degradation	Increase muscle mass and prevent muscle hypertrophy	[105]
	C2C12 cells	10 μM	FOXO3A	Decrease the level of Atrogin-1 and MuRF1	Increase muscle mass and exhibit an improvement effect on oxidative-stress-induced muscle atrophy	[107]
Betaine	C2C12 cells	10 mM	NFATc1/ MyoD signaling	Upregulate the expression of miR-29b-3p and promote myotube differentiation and the expression of slow MyHC proteins	Promote muscle cell differentiation and the transformation from fast muscle to slow muscle fiber	[136]
	C2C12 cells	10 mM	IGF-1 signaling	Increase the expression of MyoD and myogenin	Promote muscle fiber differentiation and growth	[134]
	C2C12 cells	2 or 5 mM	PGC-1α signaling	Increase mitochondrial biogenesis and ATP production	Promote muscle differentiation and the transformation from fast muscle to slow muscle fiber	[137]
Octacosanol	Male Wistar rats	2.0 μCi/dose		Promote energy mobilization and energy supply	Enhance physical performance	[140]
	Male C57BL/6 mice	200 mg/kg/d	Bcl3/TLRs/MAPK signaling	Increase the expression of Prx, Trim63 and ATPase activity	Exert an anti-fatigue effect	[143]
	Male SD rats	0.75% (*w*/*w*)	Creatine phosphorylation	Promote the shift from fast-twitch to slow-twitch myofibers	Exert an anti-fatigue effect	[141]
	Male Wistar rats	2.0 μCi/dose	ACC phosphorylation	Meet energy demand	Improve exercise capacity	[142]
β-glucan	Male SD rats	312.5 mg/kg/d		Decrease the activity of lactate dehydrogenase and the creatine kinase	Increase exercise capacity and facilitate the recovery from fatigue	[116]
	C2C12 cells	20 mg/mL	Myf5 and Mox2	Increase muscle cell proliferation and differentiation	Promote the transformation from fast muscle fibers to slow muscle fibers	[117]
	Duchenne muscular dystrophy (DMD) zebrafish model	8 mg/L	Mitochondrial respiration enzyme	Improve mitochondrial respiration and prevent oxidative stress	Improve exercise capacity	[118]
Tocotrienol	Male C57BL/6J mice	100 or 300 mg/kg/d	AMPK/SIRT1/PGC-1α signaling	Upregulate the expression of proliferation and differentiation related proteins Increase mitochondrial biogenesis	Prevent diabetes-related skeletal muscle atrophy	[110]
	Human skeletal muscle myoblasts	50 μg/mL	miR-206	Increase the expression of IGF1R and decrease the expression of Pax7	Promote muscle cell proliferation and differentiation	[111]
	Stress-induced premature senescence (SIPS) Human skeletal muscle myoblasts (CHQ5B)	50 μg/mL	Wnt signaling FOXO pathway	Downregulate the expression of MSTN and increase the expression of muscle cells regeneration related proteins such as EREG, SHC1 and SHC3	Promote muscle cell regeneration and alleviate muscle loss	[112]
	Male C57BL/6J mice	400 mg/kg/d	COXⅠ-Ⅴ	Promote mitochondrial respiration and reduce lipid peroxidation	Increase muscle mass and improve glucose homeostasis	[113]

## 4. Conclusions and Perspectives

Based on the included studies, bioactive components in whole grains exhibited important roles in regulating muscle dysfunction that can benefit individuals with related muscle diseases. Significantly, this evidence suggests that nutritional strategies are a promising way to prevent and modulate metabolic-related disorders in muscles. However, further clinical research studies are needed as there is a limited number of trials in which participants with skeletal muscle diseases are specifically targeted. Additionally, evaluation of multimodal intervention strategies such as exercise training combined with dietary supplementation, as well as the appropriate dosage and duration for the intervention are also needed which might provide novel therapeutic strategies to counteract skeletal-muscle-related diseases.

## Data Availability

Not applicable.

## Abbreviations

CSA: muscle fiber cross-sectional area; MyHC: myosin heavy chain; MD: muscular dystrophy; ATP: adenosine-triphosphate; AMPK: the adenosine monophosphate (AMP)-activated protein kinase; PGC-1a: peroxisome proliferators-activated receptor-alpha; NRF1: nuclear respiratory factor 1; TFAM: mitochondrial transcription factor A; GABP: GA binding protein transcription factor; FNIP1: folliculin interacting protein 1; mTOR: molecular target of rapamycin; mtDNA: mitochondrial DNA; ROS: reactive oxygen species; SIRT2: recombinant sirtuin 2; TGF-β: transforming growth factor-β; Smad3: mothers against DPP homolog 3; ERK: extra cellular signal-regulated kinase; FOXO3a: fork head box O3a; ERRγ: estrogen-related receptor γ; PPARδ: peroxisome proliferator-activated receptor δ; UCP3: uncoupling protein 3; MyoD: myogenic differentiation antigen; TFB2M: transcription factor B2; TNF-α: tumor necrosis factor-α; GSK-3β: glycogen synthase kinase-3β; OPCs: oligomeric procyanidins; ACC: acetyl CoA carboxylase; IGF-1: insulin-like growth factor 1; NFATc1: nuclear factor of activated T-cells; Cy3G: cyanidin-3-glucoside; Pax7: paired box gene 7; HSMM: human skeletal muscle myoblasts; SDH: succinate dehydrogenase; LDH: lactate dehydrogenase; Myf5: Myogenic determining factor 5; CK-MB: Creatine Kinase MB Isoenzyme; Mox2: Mesenchyme Homeobox 2; Prx: periaxin; Trim63: tripartite motif-containing 63; TLRs: Toll-like receptors; MAPK: mitogen-activated protein kinase; GO: Gene ontology; PKA: protein kinase A; LKB1: Liver kinase B1; NFATc1: Homo sapiens nuclear factor of activated T-cells.
